# From Biodiversity to Musketry: Detection of Plant Diversity in Pre-Industrial Peloponnese during the *Flora Graeca* Expedition

**DOI:** 10.3390/life12121957

**Published:** 2022-11-23

**Authors:** Chrysanthi Chimona, Sophia Papadopoulou, Foteini Kolyva, Maria Mina, Sophia Rhizopoulou

**Affiliations:** Section of Botany, Department of Biology, National and Kapodistrian University of Athens, 15784 Athens, Greece

**Keywords:** archives, botanical collection, Greece, landscape, pre-rebellion period

## Abstract

As the interest in natural, sustainable ecosystems arises in many fields, wild plant diversity is reconsidered. The present study is based on extant literature evidence from the journey of John Sibthorp (Professor of Botany, Oxford University) to Peloponnese (Greece) in pre-industrial time. In the year 1795, Peloponnese was a botanically unknown region, very dangerous for travellers and under civil unrest, in *conjuncture* with a pre-rebellion period. Our study reveals approximately 200 wild plant taxa that were collected from Peloponnese localities in 1795, transported to Oxford University (UK), and quoted in the magnificent edition *Flora Graeca Sibthorpiana* of the 19th century. Moreover, these plants currently constitute a living collection in Peloponnese, confirmed according to updated data on the vascular Flora of Greece. The presented lists constitute a source of information for plant biologists, linking the past to the present, shedding light on the study of adaptive traits of wild Mediterranean plants and revealing the temporal dimension of natural history. Nowadays, increasing and thorough understanding of the considered plants’ functionality to abiotic and biotic environmental stimuli provides a new framework of sustainability and management options.

## 1. Introduction

In the 18th century, travelers’ journey to Greece was also a journey through its history. The naturalists’ travels were explorations, linked to searching for specimens of natural history. The travelers’ observations became a way of identifying and revealing cultural and economic changes that have occurred over the last centuries. The botanical expeditions and the collections of specimens connected observations and descriptions with landscapes and environmental conditions; plants had been there for thousands of years, linked to the history and adapted to abiotic and biotic conditions of the localities [1,2,3,4,5].

John Sibthorp (1758–1796), Professor of Botany in the University of Oxford, decided to travel to unexplored areas of Greece, collecting and recording botanical specimens in the late 1780s and 1790s; at that time, Greece was an unknown region, very dangerous and difficult to visit owing to diseases, civil unrest, and bandit groups—known as armatoloi and klephts– that included illiterate peasants, artisans, and local clergy, together with the local notables and landowners in Peloponnese [6,7,8].

Sibthorp’s main interest was linked to plants known since the classical antiquity and mainly quoted in the texts of Dioscorides (1st century AD) [9,10,11,12,13,14]. During the first exploration from 1786 to 1787, Sibthorp was accompanied by the Austrian painter Ferdinand Lucas Bauer (1760–1826) as his draughtsman [6,7]; this was a time when travelers were accompanied by a professional artist, whose work supplemented their discoveries with visual evidence [15,16,17,18]. Actually, the magnificent, illustrated edition *Flora Graeca Sibthorpiana* (hereafter *FGS*), published from 1806 to 1840, contains botanical hand-coloured engravings that are important icons of the Mediterranean flora [7,19,20].

John Sibthorp and his companion undertook a second botanical expedition to the Levant from 1794 to 1795. During this journey, they arrived in Peloponnese (Morea is the name used in their diaries and letters) on 26 February 1795 and visited numerous localities botanizing in a more or less largely unknown area, frequently hearing the firing of guns [6,15,21,22]. Those days, major parts of Peloponnese, electively ruled by semi-autonomous agas (persons of high rank or social position during the era of the Ottoman Empire [23]), were only nominally part of the Ottoman Empire [24,25].

Although substantial, revived research has been carried out on the content of *FGS* [7,8,20,26,27,28,29,30], the Peloponnese tour and the collected botanical specimens by Sibthorp in 1795 have received little attention [6] (pp. 164–169) [7] (pp. 144–146). The importance of studying local floras, historical and environmental conditions, distribution records, and species lists has been repeatedly stressed in the literature and awareness of this subject has recently been rising.

Plants collected during a pre-rebellion period (i.e., before the Greek Revolution of 1821) in Peloponnese correspond to “visual evidence” from a particular time (spring 1795), revealing regional plant species pool of this particular area, as well as physical, cultural, and aesthetic values of the natural environment. The main goal of this study was to study plants that have been recorded in Peloponnese in pre-industrial time, as functional components of a biodiversity, which, to the best of our knowledge, has not hitherto been published. A secondary goal of this study was to confirm the above-mentioned plant diversity in Peloponnese during the 21st century.

## 2. Materials and Methods

This research is based on our survey of written sources, i.e., books, travel reports, letters, diaries, plant catalogues, online published, and printed archives mainly linked to the “Flora Graeca” expedition in Peloponnese (Greece) in 1795 [6,7]. Two copies of *FGS*, i.e., a copy adorned the National Library of Greece since 1916 and another copy acquired by the Gennadius Library of Athens in 1967 were surveyed. Moreover, we studied the digitized published hand-coloured engravings and the original watercolours, together with the Mediterranean scenes that are freely available and accessible online via Digital Bodleian (https://digital.bodleian.ox.ac.uk/collections/flora-and-fauna-graeca/, accessed on 9 October 2022). In addition, rigorous research of the *Florae Graecae Prodromus* [30] (hereafter *Prodromus*) housed in the Department of Botany at National and Kapodistrian University of Athens in Greece was carried out; it has to be noted that the *Prodromus* contains indexes of modern Greek vernacular names of plants (Index Nominum Graecorum, pp. 383–391), ancient Greek names of plants quoted in Dioscorides’ codex (Index Dioscoridem, pp. 392–404), and scientific names of plants (Index Generum et Synonymorum, pp. 405–422), as well as plant locality data [31]. Furthermore, two books were taken in consideration; the first by Robert Walpole (1781–1856, an English classical scholar with degrees from Trinity College at Cambridge in UK and Merton College at Oxford in UK, who travelled to Greece; his Memoirs including notes of various travelers’ diaries, among them Sibthorp’s and his companion [32] were first published in 1817) and the second by John Bacon Sawrey Morritt (1772–1843, who immediately after his BA degree from St. John’s College at Cambridge in UK, started on the travels described in his book that was first published in 1914; Morritt travelled over a considerable part of Peloponnese in 1795 [33]). A plant taxon was included in the results if there was a record in *Prodromus* stating locality data from Peloponnese. Information linked to the currently accepted plant nomenclature and distribution was derived from the Flora of Greece web (https://portal.cybertaxonomy.org/flora-greece/, accessed on 21 October 2022).

## 3. Results

### 3.1. Peloponnese Tour

**Figure 1:**
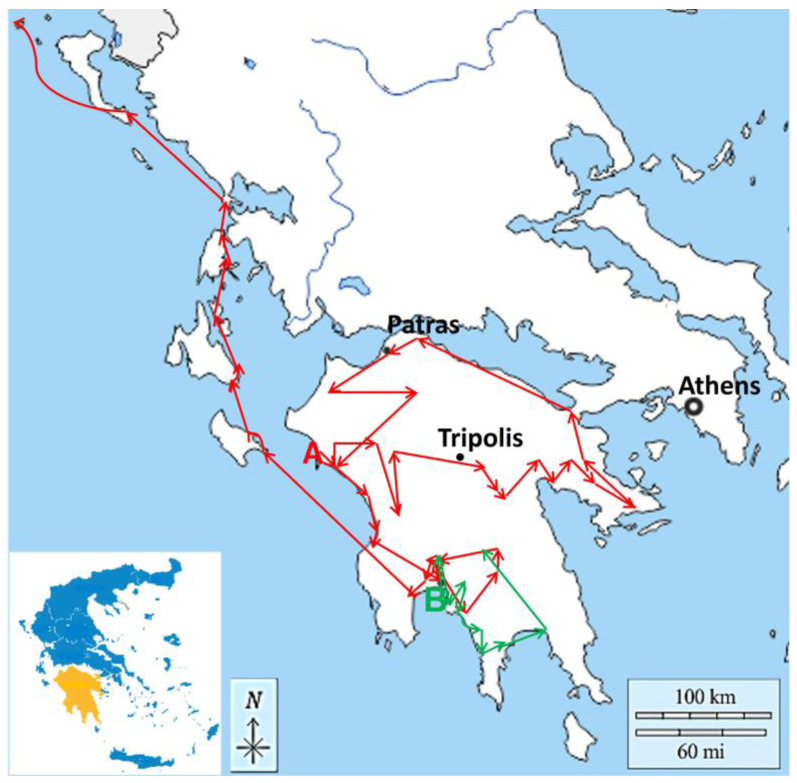
Map of Peloponnese (obtained by https://d-maps.com/ accessed on 10 October 2021 and modified accordingly), showing two tours, i.e., by Sibthorp (red line) and Morritt (green line) in 1795. The red symbol A indicates the start of Sibthorp’s journey; red lines and arrows indicate locations and directions, respectively. In the insert, the map of Greece (blue) is presented and, in yellow, the Peloponnese peninsula is indicated. The green lines and arrows indicate locations and directions of Morritt’s journey. The black-white dot indicates the capital of Greece, Athens (37.9838° N, 23.7275° E); the small black dots indicate the locations of cities: Patras (38.2466° N, 21.7346° E) and Tripolis (37.5101° N, 22.3726° E).

In Figure 1, the Peloponnese tours followed by Sibthorp and Morritt in 1795 are depicted in red and green lines, respectively. Sibthorp and his colleagues travelled from the island of Zakynthos to the port of Skaffidia (Ileia County); their route included Pyrgos, Lalla, and Tripolis, passing through several villages. The tour continued to Palaiepiscopi, ancient Tegea, and Arcadia. Next, they travelled to Argos and visited ancient Mycenae as well as Napoli di Roamin (Nafplion) in Argolida County. Then, they travelled to Korinthos and Patras, continued in Achaia County through villages, and proceeded to Ileia County again; from there, they followed different directions until they arrived in Kalamata (Messinia County). After Kalamata, they proceeded to Kutchuk Maina, Kardamili, Sparta (Laconia County), and Mystras; from there, they continued to Messini and Petallida and on 25 April 1795 they arrived at Zakynthos and, by ship, returned to England. Morritt’s journey started from Kalamata; he visited Kutchukmaina, Palaeocastro and ancient Thuria (Messinia County), Corone, Abia, and Kitreés and, through various villages, went to Kardamili/Cardamyla; he arrived by boat at Platsa and then continued to Oetylos, Marathonisi (ancient Gythium), and Mystras (Laconia County).

### 3.2. Plant Diversity in Pre-Industrial Peloponnese

Our study provides evidence for 183 plant taxa grown in pre-industrial Peloponnese, which had been collected during Sibthorp’s expedition, drawn and cited in *FGS* (Table 1). Moreover, 21 plants quoted in *Prodromus* and linked to localities of Peloponnese, but neither drawn nor cited in *FGS*, were found (Table 2). Although citations for *prickly pear* [*Opuntia ficus-indica* (L.) Mill.], walnut (*Juglans regia* L.), and mulberries (*Morus nigra* L.) were found in the considered archival research concerning Peloponnese, these plants were neither drawn nor cited in both *FGS* and *Prodromus*. It should be mentioned that the botanist Sir James Edward Smith (1759–1828)—founder and first president of the Linnean Society of London—wrote the texts for the plants attested in *FGS* and *Prodromus* and excluded all species he regarded as not being part of the natural flora.

In 1795, in western Peloponnese, *Salicornia fruticosa* L. was observed growing near lake banks, *Asphodelus ramosus* L. near rivers, and *Bromus rubens* L. in between cultivated fields. Stands of *Phillyrea latifolia* L., *Erica arborea* L., *Arbutus unedo* L., *Pistacia lentiscus* L., *vernal* (spring) *Crocus flavus* Weston, and primroses (*Primula vulgaris* Huds.) in bloom—observed in early March 1795—were encountered. In the southern Peloponnese (county of Messinia), black mulberry trees (*Morus nigra* L.) and prickly pear surrounded many villages. Moreover, they depicted fig trees (*Ficus carica* L.), grapevines, cotton, grains, corn, olive trees, *Euphorbia exigua* L., *Euphorbia spinosa* L., *Lolium perenne* L., and *Orobanche ramosa* L. Some regions produced flax and tobacco. In the eastern Peloponnese, *Quercus* species, as well as corn, grains, grapevines, olive trees, fig trees, mulberry trees, and chestnut trees, had been detected. In the central Peloponnese (county of Arcadia), they visited oaks’ forest; moreover, they observed a huge walnut tree (*Juglans regia* L.), *Hyacinthus romanus* L., and *Hyacinthus spicatus* Sm. in bloom. In addition, the presence of floating crystal-wort (*Riccia fluitans L.*) and *Boletus* (a genus of mushroom-producing fungi that comprises over 100 species) and the use of truffle were mentioned. Cultivation of pear trees with open blossoms (10 March 1795) and corns grown among the remains of cities and temples of the ancient Greek territories were detected.

John Sibthorp arrived in Peloponnese bearing a mode of seeing, endowing the professorship of “Agriculture and Rural Economy” in the University of Oxford, thus the state of the agriculture in Peloponnese attracted his attention in 1795; the cultivation of corn (*Zea mays* L.), cotton (*Gossypium hirsutum* L.), millet (*Panicum repens* L.), tobacco (*Nicotiana tabacum* L.), and wheat (*Triticum junceum* L. and *Aegilops comosa* Sm.) was detected.

## 4. Discussion

**Figure 2:**
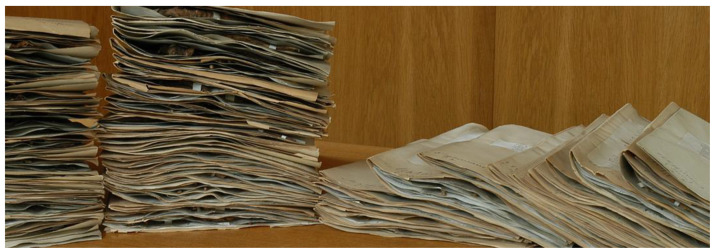
Dried specimens of plants in the Sibthorpian herbarium at the University of Oxford, associated with the Flora Graeca expeditions and collected from the eastern Mediterranean in the 18th century. Courtesy of Stephen Harris, modified by Sophia Rhizopoulou.

Professor John Sibthorp and his colleagues visited Greek territories twice in pre-industrial time, i.e., 1786–1787 and 1794–1795, and collected wild plants grown under natural conditions [7,16,34]. It was an outstanding achievement, considering the duration, the collections of specimens of plants from which “a legacy of 2462 pressed specimens are still preserved in the Sibthorpian Herbarium” [35] (Figure 2), and the geographical coverage, during the above-mentioned botanical expeditions. Moreover, a number of specimens found in Kew are of considerable importance as supplementing Sibthorp’s collection at Oxford [36]; these specimens have been published [36] according to the sequence of plants cited in *Prodromus* [30].

The revived interest in *FGS* is partially due to recent publications [22,28,37,38,39], but mainly to biodiversity issues raised under the threat of climate change, which gives another dimension to the whole achievement. Moreover, exhibitions dedicated to the concept and the content of *Flora Graeca Sibthorpiana* contributed to public awareness, e.g., in Oxford entitled “Painting by numbers” (Bodleian Library, 29 – 9 July 2017, https://treasures.bodleian.ox.ac.uk/treasures/flora-graeca/ accessed on 9 May 2017) and Athens entitled “Flora Graeca” (Gennadius Library, 8 March–4 July 2016, https://www.ascsa.edu.gr/events/details/flora-graeca-exhibition, accessed on 8 March 2016).

In Table 1, we compiled a list of 183 wild plants cited in *FGS* and located in Peloponnese, which is indicative of the biodiversity, environmental physiology, phenology, and short flowering season in response to drought conditions, i.e., during the period of spring rainfall and the concomitantly active pollinators [40,41]. The later generations of plant biologists studied plant species grown in geographic locations visited by Sibthorp and his companion in Peloponnese, increasing the overall knowledge about distribution, ecophysiology, and taxonomy of plants quoted in *FGS* and *Prodromus* [42,43,44,45,46,47,48,49]. Τhe mediterranean-type climate is characterized by a marked seasonality, typified by the alternation of a hot and dry period with a cold and wet period. For example, Sibthorp observed open flowers of *Anemone coronaria* L., *Oxalis corniculata* L., and *Asphodelus ramosus* L. on 27 February 1795, as well as of *Crocus flavus* Weston in early spring (cited as *Crocus aureus* in *FGS* and *Crocus vernus latifolius aureus* in *Prodromus*, vol. I, pp. 24–25); such observations are supported by recent publications [5,50,51]. Moreover, in the 21st century, it is known that seasonal blossom is related to adaptive floral traits; for example, the study of petals revealed a surface nano-sculpture that declines water droplet adhesion and enhances the water repellence of these fragile floral tissues, which are exposed to the rainy conditions of the early spring flowering season [52,53,54]. In *Anemone coronaria* L., the temperature plays a critical role in the onset of dormancy [55]. Other species possess deeply rooted systems that enhance drought resistance (e.g., *Myrtus communis* L., *Pistacia lentiscus* L., and *Quercus* species). In addition, recent research revealed leaf functional traits linked to hydrophobicity and water status, highlighting species’ responses to drought conditions [56,57,58]; this may be critical for resilience in the face of increasing drought stress.

Moreover, Sibthorp noticed that oaks in Peloponnese were frequently infested with the mistletoe *Loranthus europaeus* Jacq. [59,60,61]; it is worth mentioning that he regarded the deciduous, yellow *Loranthus europaeus* Jacq. as the “true mistletoe of the ancients” [6] (p. 165).

Sibthorp and his companion visited a mountainy area, barren and stony beyond conception; it was hard work botanizing under harsh field conditions. The earth, washed by the rains and torrents from the higher parts, was supported on a plethora of terraces cultivated with wheat, cotton, maize, and millet, while olives and mulberry trees seemed to grow out of the rocky substrate itself. However, carpets of geophytes and numerous annual plants produced a spring flowering distinctive to the human eye. The results from this tour in the late 1790s, in pre-industrial landscapes, barely resembled the area we see today in Peloponnese, and brought information about numerous unknown to science (those days) wild plants, oak woodlands, pine forests, crops, cultivated areas, and arable lands of the monasteries [62]. Nowadays, several places of Peloponnese that Sibthorp visited in 1795 are included in the European network Natura 2000—i.e., the cornerstone of European Union nature conservation policy—of designated sites (https://eunis.eea.europa.eu/sites, accessed on 18 October 2022) relevant for flora and habitat protection [63,64,65], e.g., mountainy landscapes such as Parnonas: GR2520006, Mainalo (Arcadia): GR2520001, and Taygetos: GR2550006, as well as Folois plateau: GR2330002 and Olympia: GR2330004. Other progression was also recorded; that is, information linked to the current distribution of the considered plants, confirmed via the Flora of Greece web, contributed to our knowledge about natural stands of wild plants.

According to our study, on one hand, among the plants found in Peloponnese in 1795 and cited in *FGS* and *Prodromus*, there are species either widely distributed or grown in restricted areas, e.g., *Achillea taygetea* Boiss. & Heldr., *Erophaca baetica* Boiss., *Saxifraga sibthorpii* Boiss., and *Scilla messeniaca* Boiss. On the other hand, *Zea mays* L., originated from the Americas and found among the few cultivated species in isolated valleys in Peloponnese in pre-industrial time, might be attributed to the Venetian occupation of Peloponnese (1688–1715); during that period, when the area was dependent on the European market, plants might have been a product of cross-cultural communication between the conquerors and conquered [66,67,68,69,70].

Sibthorp’s expedition in Peloponnese contributed to our understanding of botany in the field and revealed the diversity of plants grown in their habitats, in pre-industrial time. Historical time was linked to a gradually known plant diversity, as locations were explored and knowledge about the natural fertility of the land increased. However, anthropogenic pressure maintained by human activities, grazing, and fires in Peloponnese added to environmental stresses and caused profound transformation in the natural landscape, reducing the distribution of indigenous plants and enhancing a widespread concern about the extent of habitat and species loss [71,72,73,74,75,76]. This means that whatever effort can be made to study, maintain, and protect the diversity of ecosystems in this region is closely connected to a sustainable future, via the preservation of numerous plant taxa cited in the monumental *FGS* and *Prodromus*. Nowadays, Oxford Botanic Garden in UK (where visitors can enjoy the full sensory experience of walking through an aromatic Mediterranean landscape while learning about the work of Sibthorp and Bauer and its important botanical and horticultural legacy [35]) and Diomedes’ Botanic Garden in Greece (due to the fact that administration of Diomedes’ Botanic Garden is directly linked to the staff of the National and Kapodistrian University of Athens in Greece, this Garden has also been used for relevant, educational programs [37]) contain living collections of Mediterranean plants cited in *FGS*, which may be perceived as celebrations for Flora Graeca expeditions and *FGS* [35,37]. However, a larger number of plants quoted in *FGS* and *Prodromus* may be introduced and cultivated in the above-mentioned botanic gardens and/or the network of botanic gardens in Greece, in order to detect the diversity and the life-cycle of wild plants within the context of the seasons, floral colours in Mediterranean ecosystems, and collection and deposition of seeds in seed-banks. As such, botanic gardens can be used as common gardens, where researchers can conduct unmatched comparative research studies of plant ecophysiology, morphology, anatomy, and responses to climate change [77,78]. It is worth mentioning that Sibthorp introduced new species into English horticulture; moreover, he returned to Oxford from his eastern Mediterranean explorations with seeds, bulbs, and corms for the Botanic Garden, but few details of these collections have survived, and the plants and any knowledge about their propagation have been lost through many routes [7] (p. 180) and neglected [79] (p. 102).

This work provides a novel and valuable insight into the development of early plant environmental biology and is an important element of timelessness aspects of botany [80,81]. The study of plant diversity in Peloponnese peninsula, during the pre-rebellion period in Greece, tracing long-term changes in the region, is also a reminder that nature is often a repository at which nations look when crafting their identity.

## 5. Conclusions

The interest in archival material has been revived on account of research for a biodiversity threatened by climatic change. In this context, our research gives prominence to approximately 200 wild plant taxa found in Peloponnese (Greece)—most of them quoted in the magnificent edition *Flora Graeca Sibthorpiana* of the 19th century—and few cultivated introduced plants, all grown under ambient conditions and exposed to environmental stresses of the eastern Mediterranean during the pre-rebellion period, representing plant environmental issues in pre-industrial time, which have not hitherto been published.

## Figures and Tables

**Table 1:** List of plants found in Peloponnese and cited in *Flora Graeca Sibthorpiana* (*FGS*). First column: plant names quoted in the first edition of *FGS* (1806–1840). Second column: numerical register of hand-coloured engravings (plates) of plants cited in the first published edition of *FGS*. Third column: numerical register of the original watercolours by Ferdinand Bauer preserved at Oxford (MS. Sherard 241–245), digitized and electronically accessed via Digital Bodleian; whenever the picture of the original drawing was not digitally available, the digital hand-coloured engraving from the first printed edition is mentioned (Sherard 761 and 764). Fourth column: current scientific name.

Plant Name Cited in *FGS*	Engraving	Watercoulor	Scientific Name
*Phillyrea* *latifolia*	2	761: pl.2	*Phillyrea latifolia* L.
*Olea europaea*	3	761: pl.3	*Olea europaea* L.
*Veronica glauca*	7	244: f.66	*Veronica glauca* Sm.
*Veronica triphyllos*	10	244: f.69	*Veronica triphyllos* L.
*Salvia triloba*	17	244: f.158	*Salvia fruticosa* Mill.
*Salvia ringens*	18	244: f.159	*Salvia ringens* Sm.
*Salvia sibthorpii*	22	244: f.163	*Salvia virgata* Jacq.
*Morina persica*	28	761: pl.28	*Morina persica* L.
*Crocus aureus*	35	245: f.65	*Crocus flavus* Weston subsp. *flavus*
*Iris florentina*	39	245: f.69	*Iris albicans* Lange
*Iris sisyrinchium*	42	245: f.72	*Moraea sisyrinchium* (L.) Ker-Gawl.
*Schoenus mucronatus*	43	245: f.112	*Cyperus capitatus* Vand.
*Saccharum ravennae*	52	245: f.120	*Tripidium ravennae* (L.) H. Scholz
*Panicum repens*	61	245: f.130	*Panicum repens* L.
*Briza minor*	74	245: f.142	*Briza minor* L.
*Festuga littoralis*	80	245: f.148	*Aeluropus littoralis* (Gouan) Parl.
*Bromus tectorum*	82	245: f.150	*Bromus tectorum* L.
*Bromus rubens*	83	245: f.151	*Bromus rubens* L.
*Stipa paleacea*	86	245: f.154	*Stipa capensis* Thunb.
*Triticum* *junceum*	99	245: f.166	*Elytrigia juncea* (L.) Nevski
*Valantia muralis*	137	242: f.202	*Valantia muralis* L.
*Crucianella latifolia*	139	242: f.204	*Crucianella latifolia* L.
*Plantago lagopus*	144	244: f.182	*Plantago lagopus* L.
*Hypecoum imberbe*	156	241: f.30	*Hypecoum imberbe* Sm.
*Anchusa tinctoria*	166	244: f.33	*Anchusa tinctoria* L.
*Cerinthe aspera*	170	244: f.37	*Cerinthe major* L.
*Cerinthe retorta*	171	244: f.38	*Cerinthe retorta* Sm.
*Asperugo procumbens*	177	244: f.44	*Asperugo procumbens* L.
*Lycopsis variegata*	178	244: f.36	*Anchusella variegata* (L.) Bigazzi & al.
*Primula vulgaris*	184	244: f.175	*Primula vulgaris* Huds.
*Lysimachia linum-stellatum*	189	244: f.181	*Asterolinon linum-stellatum* (L.) Duby
*Plumbago europaea*	191	244: f.196	*Plumbago europaea* L.
*Convolvulus siculus*	196	244: f.15	*Convolvulus siculus* L.
*Campanula rupestris*	213	243: f.178	*Campanula rupestris* Sm.
*Campanula drabifolia*	215	243: f.180	*Campanula drabifolia* Sm.
*Viola gracilis*	222	241: f.85	*Viola gracilis* Sm.
*Chironia maritima*	237	244: f.9	*Centaurium maritimum* (L.) Fritsch
*Chironia spicata*	238	244: f.10	*Schenkia spicata* (L.) G. Mans.
*Vitis vinifera*	242	241: f.178	*Vitis vinifera* L.
*Herniaria macrocarpa*	252	242: f.125	*Herniaria incana* Lam.
*Eryngium multifidum*	259	242: f.148	*Eryngium amethystinum* L.
*Bupleurum sibthorpianum*	264	242: f.153	*Bupleurum falcatum* subsp. *cernuum* (Ten.) Arcang.
*Echinophora spinosa*	265	242: f.154	*Echinophora spinosa* L.
*Echinophora tenuifolia*	266	242: f.155	*Echinophora tenuifolia* L.
*Artedia squamata*	268	242: f.157	*Artedia squamata* L.
*Peucedanum obtusifolium*	277	242: f.175	*Selinum silaifolium* (Jacq.) Beck
*Coriandrum sativum*	283	242: f.170	*Coriandrum sativum* L.
*Pastinaca opopanax*	288	242: f.176	*Opopanax hispidus* (Friv.) Griseb.
*Linum gallicum*	303	241: f.160	*Linum trigynum* L.
*Narcissus tazetta*	308	245: f.73	*Narcissus tazetta* L.
*Amaryllis lutea*	310	245: f.75	*Sternbergia lutea* (L.) Spreng. subsp. *lutea*
*Tulipa sibthorpiana*	330	245: f.79	*Fritillaria sibthorpiana* (Sm.) Baker
*Ornithogalum arvense*	332	245: f.97	*Gagea villosa* (M. Bieb.) Sweet
*Ornithogalum nanum*	333	245: f.98	*Ornithogalum sibthorpii* Greuter
*Asphodelus ramosus*	334	245: f.99	*Asphodelus ramosus* L.
*Anthericum graecum*	336	245: f.101	*Gagea graeca* (L.) Irmisch
*Asparagus acutifolius*	337	245: f.102	*Asparagus acutifolius* L.
*Hyacinthus romanus*	340	245: f.105	*Bellevalia romana* (L.) Sweet
*Frankenia hirsuta*	343	241: f.88	*Frankenia hirsuta* L.
*Erica arborea*	351	243: f.190	*Erica arborea* L.
*Arbutus unedo*	373	243: f.191	*Arbutus unedo* L.
*Arbutus andrachne*	374	243: f.192	*Arbutus andrachne* L.
*Saxifraga* *media*	376	242: f.142	*Saxifraga sempervivum* K. Koch
*Saxifraga rotundifolia*	377	242: f.143	*Saxifraga rotundifolia* L.
*Saxifraga cymbalaria*	378	242: f.144	*Saxifraga sibthorpii* Boiss.
*Dianthus cinnamomeus*	400	241: f.110	*Dianthus cinnamomeus* Sm.
*Silene nocturna*	408	241: f.118	*Silene nocturna* L.
*Silene behen*	416	241: f.126	*Silene behen* L.
*Silene italica*	429	241: f.138	*Silene italica* (L.) Pers.
*Silene staticifolia*	434	241: f.144	*Silene bupleuroides* subsp. *staticifolia* (Sm.) Chowdhuri
*Sedum tetraphyllum*	448	242: f.135	*Sedum cepaea* L.
*Oxalis corniculata*	451	241: f.190	*Oxalis corniculata* L.
*Cerastium pilosum*	454	241: f.149	*Cerastium illyricum* Ard.
*Cerastium tomentosum*	455	241: f.150	*Cerastium candidissimum* Correns
*Reseda alba*	459	245: f.49	*Reseda alba* L.
*Euphorbia spinosa*	463	245: f.39	*Euphorbia acanthothamnos* Boiss.
*Euphorbia leiosperma*	465	245: f.41	*Euphorbia terracina* L.
*Myrtus communis*	475	242: f.120	*Myrtus communis* L.
*Prunus prostrata*	478	242: f. 109	*Prunus prostrata* Labill.
*Pyrus aria*	479	242: f.118	*Sorbus umbellata* (Desf.) Fritsch
*Papaver somniferum*	491	241: f.24	*Papaver somniferum* L.
*Cistus monspeliensis*	493	241: f.75	*Cistus monspeliensis* L.
*Cistus incanus*	494	241: f.74	*Cistus creticus* subsp. *eriocephalus* (Viv.) Greuter & Burdet
*Cistus salviifolius*	497	241: f.78	*Cistus salviifolius* L.
*Cistus guttatus*	498	241: f.79	*Tuberaria guttata* (L.) Fourr.
*Cistus salicifolius*	499	241: f.80	*Helianthemum salicifolium* (L.) Mill.
*Delphinium consolida*	504	241: f.7	*Consolida phrygia* (Boiss.) Soó
*Anemone coronaria*	514	241: f.17	*Anemone coronaria* L.
*Ranunculus millefoliatus*	521	241: f.4	*Ranunculus millefoliatus* Vahl
*Satureja juliana*	540	244: f.117	*Micromeria juliana* (L.) Rchb.
*Satureja graeca*	542	244: f.118	*Micromeria graeca* (L.) Rchb.
*Satureja capitata*	544	244: f.115	*Thymbra capitata* (L.) Cav.
*Nepeta nuda*	547	244: f.120	*Nepeta nuda* L.
*Lamium maculatum*	556	244: f.127	*Lamium maculatum* L.
*Stachys orientalis*	560	244: f.146	*Stachys obliqua* Waldst. & Kit.
*Marrubium pseudodictamnus*	562	244: f.147	*Ballota pseudodictamnus* (L.) Benth.
*Prasium majus*	584	244: f.155	*Prasium majus* L.
*Bartsia latifolia*	586	244: f.71	*Bellardia latifolia* (L.) Cuatrec.
*Antirrhinum pelisserianum*	591	244: f.76	*Linaria pelisseriana* (L.) Mill.
*Antirrhinum chalepense*	592	244: f.77	*Linaria chalepensis* (L.) Mill.
*Antirrhinum reflexum*	593	244: f.78	*Linaria triphylla* (L.) Mill.
*Scrophularia canina*	598	244: f.83	*Scrophularia canina* subsp. *bicolor* (Sm.) Greuter
*Scrophularia caesia*	604	244: f.89	*Scrophularia heterophylla* Willd.
*Orobanche ramosa*	608	244: f.93	*Phelipanche mutelii* (F.W. Schultz) Pomel
*Acanthus spinosus*	611	244: f.95	*Acanthus spinosus* L.
*Bunias raphanifolia*	612	241: f.33	*Rapistrum rugosum* (L.) All.
*Aubrieta deltoidea*	628	241: f.49	*Aubrieta deltoidea* (L.) DC.
*Biscutella columnae*	629	241: f.50	*Biscutella didyma* subsp. *apula* Nyman
*Arabis verna*	641	241: f.62	*Arabis verna* (L.) R. Br.
*Erodium romanum*	654	241: f.182	*Erodium acaule* (L.) Bech. & Thell.
*Erodium gruinum*	656	241: f.184	*Erodium gruinum* (L.) L’Hér.
*Erodium malacoides*	658	241: f.186	*Erodium malacoides* (L.) L’Hér.
*Geranium tuberosum*	659	241: f.187	*Geranium tuberosum* L.
*Alcea ficifolia*	663	241: f.166	*Alcea biennis* Winterl
*Hibiscus trionum*	666	241: f.169	*Hibiscus trionum* L.
*Polygala venulosa*	669	241: f.86	*Polygala venulosa* Sm.
*Ononis antiquorum*	675	242: f.11	*Ononis spinosa* subsp. *diacantha* (Rchb.) Greuter
*Anthyllis tetraphylla*	681	242: f.17	*Tripodion tetraphyllum* (L.) Fourr.
*Orobus sessilifolius*	692	242: f.27	*Lathyrus digitatus* (M. Bieb.) Fiori
*Lathyrus sativus*	695	242: f.31	*Lathyrus sativus* L.
*Lathyrus grandiflorus*	698	242: f.34	*Lathyrus grandiflorus* Sm.
*Vicia polyphylla*	699	242: f.35	*Vicia villosa* subsp. *varia* (Host) Corb.
*Vicia melanops*	701	242: f.37	*Vicia melanops* Sm.
*Cytisus sessilifolius*	705	242: f.41	*Podocytisus caramanicus* Boiss. & Heldr.
*Coronilla emerus*	710	242: f.46	*Hippocrepis emerus* (L.) Lassen
*Coronilla securidaca*	712	242: f.48	*Securigera securidaca* (L.) Degen & Dörfl.
*Ornithopus compressus*	714	242: f.50	*Ornithopus compressus* L.
*Ornithopus scorpioides*	715	242: f.51	*Coronilla scorpioides* (L.) W.D.J. Koch
*Hippocrepis unisiliquosa*	716	242: f.52	*Hippocrepis unisiliquosa* L.
*Hedysarum c* *aput* *-galli*	723	242: f.59	*Onobrychis caput-galli* (L.) Lam.
*Phaca baetica*	727	242: f.63	*Erophaca baetica* (L.) Boiss.
*Astragalus incanus*	732	242: f.68	*Astragalus spruneri* Boiss.
*Astragalus aristatus*	735	242: f.71	*Astragalus thracicus* subsp. *parnassi* (Boiss.) Strid
*Biserrula pelecinus*	737	242: f.73	*Astragalus pelecinus* (L.) Barneby
*Trifolium cherleri*	745	242: f.81	*Trifolium cherleri* L.
*Trifolium rotundifolium*	747	764: pl.747	*Trigonella rotundifolia* (Sm.) Strid
*Trifolium stellatum*	750	242: f.86	*Trifolium stellatum* L.
*Trifolium clypeatum*	751	242: f.87	*Trifolium clypeatum* L.
*Trifolium uniflorum*	752	242: f.88	*Trifolium uniflorum* L.
*Lotus tetragonolobus*	755	242: f.91	*Tetragonolobus purpureus* Moench
*Lotus edulis*	756	242: f.92	*Lotus edulis* L.
*Lotus creticus*	758	242: f.94	*Lotus creticus* L.
*Lotus hirsutus*	759	242: f.95	*Dorycnium hirsutum* (L.) Ser.
*Trigonella corniculata*	761	242: f.97	*Trigonella corniculata* (L.) L.
*Trigonella monspeliaca*	765	242: f.101	*Medicago monspeliaca* (L.) Trautv.
*Medicago marina*	770	242: f.106	*Medicago marina* L.
*Hypericum olympicum*	772	241: f.171	*Hypericum olympicum* L.
*Hypericum hircinum*	773	241: f.172	*Hypericum hircinum* L.
*Hypericum crispum*	776	241: f.175	*Hypericum triquetrifolium* Turra
*Scorzonera laciniata*	788	243: f.144	*Podospermum laciniatum* (L.) DC.
*Sonchus picroides*	793	243: f.166	*Reichardia picroides* (L.) Roth
*Crepis rubra*	801	243: f.157	*Crepis rubra* L.
*Hedypnois cretica*	813	243: f.132	*Hedypnois rhagadioloides* (L.) F.W. Schmidt
*Hypochoeris minima*	816	243: f.123	*Hypochaeris arachnoides* Poir.
*Lapsana stellata*	817	243: f.126	*Rhagadiolus stellatus* (L.) Gaertn.
*Catananche lutea*	821	243: f.129	*Catananche lutea* L.
*Carduus glycacanthus*	826	243: f.96	*Jurinea glycacantha* DC.
*Cnicus acarna*	827	243: f.94	*Picnomon acarna* (L.) Cass.
*Onopordum elatum*	833	243: f.87	*Onopordum tauricum* Willd.
*Cynara humilis*	835	243: f.89	*Cynara cardunculus* L.
*Carlina lanata*	836	243: f.82	*Carlina lanata* L.
*Carlina corymbosa*	837	243: f.83	*Carlina corymbosa* subsp. *graeca* (Heldr. & Sartori) Nyman
*Acarna cancellata*	839	243: f.85	*Atractylis cancellata* L.
*Carthamus lanatus*	841	243: f.118	*Carthamus lanatus* L.
*Carthamus caeruleus*	843	243: f.120	*Carthamus caeruleus* L.
*Staehelina chamaepeuce*	847	243: f.90	*Ptilostemon chamaepeuce* (L.) Less.
*Senecio trilobus*	869	243: f.65	*Senecio trilobus* L.
*Bellis annua*	876	243: f.22	*Bellis annua* L.
*Chrysanthemum coronarium*	877	243: f.58	*Glebionis coronaria* (L.) Spach
*Anthemis cota*	880	243: f.35	*Anthemis altissima* L.
*Anthemis altissima*	881	243: f.36	*Anthemis altissima* L.
*Achillea aegyptiaca*	892	243: f.51	*Achillea taygetea* Boiss. & Heldr.
*Centaurea benedicta*	906	243: f.114	*Centaurea benedicta* (L.) L.
*Centaurea aegyptiaca*	907	243: f.102	*Centaurea aegyptiaca* Sm.
*Centaurea melitensis*	909	243: f.104	*Centaurea melitensis* L.
*Centaurea collina*	914	243: f.109	*Centaurea salonitana* Vis.
*Centaurea galactites*	919	243: f.115	*Galactites tomentosus* Moench
*Filago pygmaea*	921	243: f.28	*Filago pygmaea* L.
*Orchis undulatifolia*	927	245: f.58	*Orchis italica* Poir.
*Orchis papilionacea*	928	245: f.59	*Anacamptis papilionacea* subsp. *aegaea* (P. Delforge) L. Lewis & Kreutz
*Ophrys fusca*	930	245: f.61	*Ophrys fusca* Link
*Pistacia terebinthus*	956	242: f.4	*Pistacia terebinthus* L.
*Atriplex halimus*	962	245: f.8	*Atriplex halimus* L.

**Table 2:** List of plants found in Peloponnese and cited in *Prodromus*. First column: plant names alphabetically presented according to the name given in archives, which are quoted in *Prodromus*, but not referred in *FGS*. Second column: numerical register of volume and page, respectively, in *Prodromus*. Third column: current scientific name.

Plant Name Cited in *Prodromus*	Volume, Page	Scientific Name
*Castanea sativa*	2, 242	*Castanea sativa* Mill.
*Corylus* spp. (hazel)	2, 244	*Corylus avellana* L., *C. colurna* L.
*Euphorbia apios*	1, 326	*Euphorbia apios* L.
*Ficus carica*	2, 268	*Ficus carica* L.
*Fraxinella*	1, 271	*Dictamnus albus* L.
*Globularia alypum*	1, 78	*Globularia alypum* L.
*Leontic* *e* *altaica*	1, 234	*Gymnospermium peloponnesiacum* (Phitos) Strid
*Leontice chrysogonum*	1, 234	*Bongardia chrysogonum* (L.) Spach
*Leontice leontopetalum*	1, 234	*Leontice leontopetalum* L.
*Lolium*	1, 70	*Lolium perenne* L., *L. subulatum* Vis., *L. temulentum* L.
*Imperatoria*	1, 199	*Imperatoria ostruthium* L.
*Loranthus*	1, 242	*Loranthus europaeus* Jacq.
*Urtica*	2, 233	*Urtica dioica* L., *U. pilulifera* L., *U. urens* L.
*Quercus* spp.	2, 239	*Quercus aegilops* L., *Q. coccifera* L., *Q. ilex* L., *Q. pubescens* Willd.
*Pinus*	2, 242	*Pinus pinea* L.
*Rubus* spp.	1, 349	*Rubus sanctus* Schreb., *R*. *canescens* DC.
*Salicornia*	1, 1	*Salicornia fruticosa* L., *S. perennans* Willd.
*Satyrium*	2, 215	*Satyrium* L., *Orchis* sp.
*Scilla*	1, 237	*Scilla nivalis* Boiss., *S. messeniaca* Boiss., *S. pneumonanthe* Speta
*Viola*	1, 145	*Viola scorpiuroides* Coss., *Viola graeca* (W. Becker) Halácsy
*Nymphaea*	1, 360–361	*Nymphaea alba* L.

## Data Availability

The data are available from the authors upon request.
